# Ventriculoperitoneal shunt for giant porencephaly: a case report and literature review

**DOI:** 10.3389/fsurg.2024.1389050

**Published:** 2024-04-19

**Authors:** Dongsheng Lu, Jialiang Tan, Haitong Xu

**Affiliations:** Department of Neurosurgery, Guangdong Sanjiu Brain Hospital, Guangzhou, China

**Keywords:** porencephaly (POR), epilepsy, cerebrospinal fluid (CSF), magnetic resonance imaging, ventriculoperitoneal shunt, case report

## Abstract

Porencephaly (POR) is an exceedingly rare neurological disorder characterized by the presence of solitary or multiple regressive cerebrospinal fluid (CSF) cavities within the brain parenchyma. Currently, there is a limited understanding of the pathogenesis and treatment options for this condition, and clinical presentations can vary significantly. However, imaging plays a crucial role in diagnosis and determining the optimal treatment strategy, necessitating individualized comprehensive treatment upon detection. We reported a 25-year-old male case with persistent head pain that did not resolve with rest. Magnetic resonance imaging (MRI) confirmed the giant POR, and we finally performed a ventriculoperitoneal shunt, and the symptoms of intracranial hypertension were relieved after surgery.

## 1 Introduction

Porencephaly (POR) is an extremely rare congenital brain disorder, characterized by a cerebrospinal fluid-filled cavity within the brain parenchyma. It can be congenital or acquired as a result of infection, trauma, infarction, or hemorrhage (1–3). The pathogenesis remains unclear (4–7). POR can occur in any cerebral lobe, usually unilateral, with bilateral hemisphere involvement being an exceedingly rare manifestation. Clinical presentations of POR are diverse, possibly related to the lesion's location and size (8, 9). Cranial magnetic resonance imaging (MRI) is the gold standard for diagnosing POR. Treatment options for POR and its potential complications include physical therapy for neurological deficits, antiepileptic drugs for seizure control, and shunting for hydrocephalus. However, surgical intervention is recommended for cases resistant to antiepileptic medication (1, 8, 9). Therefore, individualized comprehensive treatment should be tailored to each patient's specific condition. We report the case of a 25-year-old male patient with imaging studies showing a giant POR occupying almost the entire right hemisphere, presenting with symptoms of intracranial hypertensive headache and epilepsy, treated with a combination of ventriculoperitoneal shunt surgery and antiepileptic drugs. Finally, we provide a brief description of the literature on POR.

## 2 Clinical presentation

On October 27, 2023, a 25-year-old man was hospitalized due to enduring headaches that had begun abruptly 20 days prior. The headache, which started without any apparent trigger, is characterized by continuous swelling pain predominantly in the left frontal-temporal area and does not subside with rest. There are no accompanying symptoms such as dizziness, nausea, vomiting, or limb convulsions; his urination and defecation are normal. A cranial MRI performed at a local hospital revealed a POR, but conservative treatment failed to alleviate the headache; hence, he was admitted for further diagnosis and treatment. Imaging studies conducted at two months of age indicated underdevelopment of the right cerebral hemisphere, though detailed specifics remain unclear. Over ten years ago, he began experiencing sudden epileptic episodes, manifesting as periods of staring blankly with loss of consciousness, resolving spontaneously after approximately 3–5 min and occurring around every two days. Following an epilepsy diagnosis, he commenced treatment with Carbamazepine, which reduced the frequency of episodes to about once per month. Two years ago, his medication regimen was adjusted, maintaining the seizure frequency at roughly once per month. Upon this discharge, his medication doses were again adjusted (Sodium Valproate Sustained Release Tablets 500 mg/dose, twice daily, and Topiramate Tablets 50 mg/dose, twice daily), yet this did not fully control the seizure frequency. He has no history of surgery, no abnormalities in the perinatal period, was born full-term via normal delivery, from a non-consanguineous marriage, with no family history of epilepsy. Physical examination upon admission showed a normal head circumference, right-hand dominance, clear consciousness, coherent speech, and slight cognitive and intellectual decline, with no psychiatric abnormalities and no other anomalies noted.

**Figure 1:**
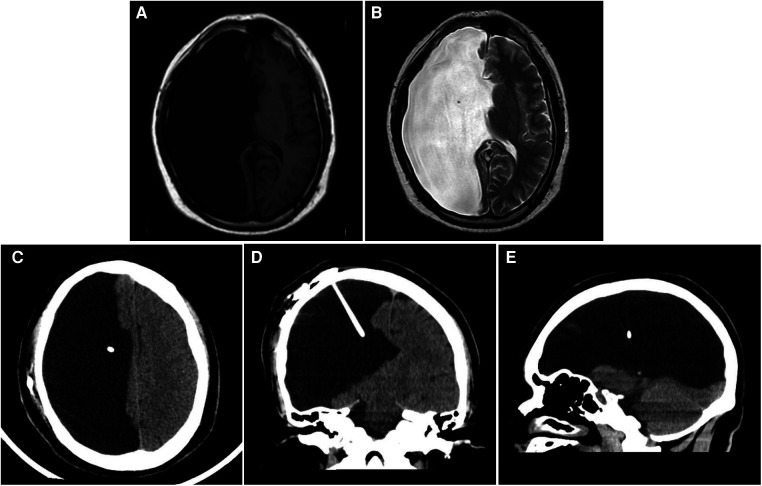
Imaging examination. (**A,B**) Cranial T1(**A**) and T2(**B**) MR images of the brain demonstrating a massive right intracranial cyst with CSF density. (**C–E**) Cranial CT scan indicates the position of the end of the postoperative shunt. (**C**) transverse section; (**D**) coronal section; (**E**) sagittal section.

Combining cranial MRI plain scan, CSF flow sequence, and cranial CT examination suggested extensive softening lesions and developmental abnormalities in the right cerebral hemisphere, accompanied by POR (Figures 1A,B). In electroencephalogram (EEG) examination, asymmetry in the background activity of both brain regions was first noted, followed by epileptic discharges in the left temporal region during interictal periods, and slow wave activity with medium amplitude starting in the left temporal region during ictal periods; two clinical seizure events were recorded via video monitoring: the patient exhibited unconscious autonomous activities in the right hand, followed by muscle tone disorders in the left upper limb. Electrocardiogram, chest CT, and ultrasound examinations of the liver, gallbladder, pancreas, and spleen showed no significant abnormalities. The neuropsychological assessment indicated a mild decline in cognition and intelligence. Preoperative routine blood tests, liver function, kidney function, and electrolytes revealed no notable abnormalities. After a multidisciplinary consultation, the patient underwent a lumbar puncture for a fluid release experiment, followed by two lumbar punctures for pressure measurement and fluid release, after which the patient reported significant relief from headache symptoms compared to before. The initial pressures of both procedures were approximately 220 mmH2O, with final pressures around 90 mmH2O; meanwhile, routine, biochemical, and cytological analyses of the CSF showed no significant abnormalities. Based on the patient's clinical presentation and auxiliary examinations, a shunting procedure was ultimately performed using a second-generation adjustable anti-magnetic shunt tube (Aesculap, Braun, Germany). Then the pressure was adjusted to 150 mmH2O, and postoperative cranial CT confirmed the good placement of the drainage tube (Figures 1C–E). The patient experienced significant alleviation in symptoms of head discomfort from the second day after anesthesia recovery. On the 11th postoperative day, the patient was discharged with improved headache symptoms. We conducted short-term follow-up of the patients at 3 and 5 months after surgery, and no pathological symptoms such as headache, nausea and vomiting, unsteady walking, and urinary incontinence were observed while the shunt valve pressure remained unchanged, and the results of physical examination and neuropsychological evaluation were similar to those before surgery. On the other hand, under the premise of ensuring continuous oral antiepileptic drugs, the frequency and form of seizures did not change significantly from before surgery.

## 3 Discussion

We report a case of a patient with a giant POR with seizures who received surgical treatment, followed by a literature review on POR (1).

### 3.1 Epidemiology

Hino-Fukuyo et al. found that the overall prevalence of POR in Asian populations was approximately 5.3 per 100,000 live births (2). Several studies in the United States and European countries suggest that the prevalence of POR at birth is 3.5 per 100,000 live births (3). Therefore, this phenomenon indicates that the prevalence of POR is rare worldwide and the survival rate is low.

### 3.2 Pathogenesis

Some expert studies believe that most cases of POR are caused by vascular lesions, which is a type of post-migratory stroke, which causes the area of tissue necrosis within the fissure to be absorbed, leaving a lesion of a foramen cyst (4, 5). In rare cases, infection or traumatic injury may also lead to the development of POR, and there have even been reports of a pregnant woman with carbon monoxide poisoning resulting in a child born with a POR (5). In this case, we found that the patient had left upper side weakness and unsteady holding at 2 months of age, and there was no abnormality in the perinatal history. Then the complete brain MRI showed that the right cerebral hemisphere was absent, and no cerebral gray matter tissue was found in the brain fissure, and the missing side was a cyst filled with cerebrospinal fluid. Therefore, we consider that this patient is likely to have stroke symptoms following brain neuron migration, which in turn leads to the formation of POR.

On the other hand, a small number of experts believe that there is phenotypic diversity in COL4A1 mutations, which is one of the important causes of POR. However, COL4A1 mutations can cause not only familial cases of POR, but also a large proportion (approximately 21%) of sporadic cases (6, 7). Therefore, in the process of genetic counseling and examination of POR, once there is a case related to COL4A1 mutation, we need to pay more attention to its family heritability.

### 3.3 Clinical presentation

POR can occur in any cerebral lobe, but is typically unilateral, with bilateral POR being even rarer; moreover, the clinical manifestations of POR vary significantly depending on their size, ranging from small focal lesions within a lobe to massive lesions occupying an entire cerebral hemisphere (8, 9). When POR occupies the temporal lobe or a region of the hippocampus or leads to conditions such as hippocampal sclerosis, epileptic seizures are among the most likely clinical presentations to occur (10). Researchers have also found that patients with POR may experience various types of epileptic seizures, mental illnesses (such as intellectual disability, delusions, apathy, or persecution complex), motor disorders (including hemiparesis, limb spasticity, or ataxia), as well as diplopia and cerebrospinal fluid leakage among other conditions, with different types of epileptic seizures being the most common. Consequently, some patients are diagnosed with this congenital disorder only after being admitted to the hospital for epileptic seizures (11–13). In this case, imaging revealed a large POR on the right side; the patient first experienced epileptic seizures around the age of ten. Additionally, as the patient aged, there was a gradual decline in cognitive functions and intelligence; these symptoms indicate that the presence of abnormal neurodevelopment in POR may have a pathologically plastic effect on symptom presentation.

### 3.4 Auxiliary examination and differential diagnosis

Cranial MRI serves as the gold standard for diagnosing POR. Within cranial MRI images, not only can the location of POR abnormalities be precisely identified, but it is also possible to distinguish whether these are cysts filled with cerebrospinal fluid or the formation of partial cavities. The primary manifestation of POR in most patients involves cystic lesions within the brain, which, across all cranial MRI sequences, adhere to the signal characteristics of CSF and demonstrate connectivity with the ventricular system or subarachnoid space (1, 8, 14). Moreover, in the process of managing epileptic seizures, cognitive function alterations, and other abnormal clinical manifestations caused by POR, specialized examinations such as PET-CT, long-term video EEG monitoring, Wechsler Memory Scale, Wechsler Intelligence Scale, and the Montreal Cognitive Assessment can be utilized. Based on clinical manifestations and auxiliary examinations, a comprehensive assessment of the impact POR has on patient development, as well as the extent of potential remediation and intervention, can be summarized. On the other hand, sequence analysis via cranial MRI also facilitates further differential diagnosis with related diseases such as schizencephaly, neuroglial cyst, arachnoid cyst, and holoprosencephaly, thereby clarifying the diagnosis of POR and devising the optimal individualized treatment plan (14, 15).

### 3.5 Treatment

Since the clinical symptoms caused by the POR depend on the location and size of the malformation, the clinical symptoms are varied, and the treatment methods vary from person to person. Ho et al. showed that the seizures caused by the POR were common, and the treatment was based on the evaluation of the temporal lobe and hippocampal structures by various MRI sequences and PET-CT, followed by 24-h video electroencephalogram monitoring, and finally, antiepileptic drug therapy and further temporal lobe epileptic resection, which showed that the treatment of epilepsy caused by the POR was effective (16). In this case, we found that the patient's initial clinical manifestation was generalized seizures, and oral antiepileptic drugs were used to control the number and duration of seizures. The 24-h video EEG after admission revealed extensive abnormalities in the left temporal lobe during the clinical seizure period, so it was difficult to choose the best surgical option to continue the symptomatic treatment with oral antiepileptic drugs.

On the other hand, the symptoms of intracranial hypertension gradually appeared in the process of development and growth, and the headache symptoms were significantly relieved after the fluid release experiment. Based on previous reports of hydrocephalus caused by POR (13, 17), combined with the patient's brain MRI showing that the porencephaly cyst communicates with the ventricular system, to alleviate the symptoms of intracranial hypertension, it was decided to perform shunt surgery for the patient. The patient's intracranial hypertension symptoms were significantly alleviated after surgery. However, the key to shunt surgery is the regulation of shunt valve pressure. Because the main feature of POR is that there is no gray matter lesion in the fissure, when the pressure is too high, it cannot achieve the effect of reducing intracranial pressure. When the pressure is too low, it will lead to tearing and bleeding of perforated malformed blood vessels, and even lead to the formation of brain herniation, and life-threatening. Therefore, for the adjustment of the shunt valve, on the one hand, the patient's clinical symptoms can be taken as one of the observation points to see whether the symptoms of the patient's intracranial hypertension headache are relieved after pressure adjustment. On the other hand, cranial CT or MRI can be used to evaluate changes in porencephaly cysts and changes in compression or exudation of peripheral brain tissue (18). For diseases such as POR and epilepsy, we not only provide individualized comprehensive treatment but also improve the quality of life of patients.

## 4 Conclusion

POR is a relatively rare central nervous system disorder, with insidious onset. The variations in condition and clinical manifestations are somewhat correlated with the malformation's location and size. Hence, clinical presentations vary, with epileptic seizures and hydrocephalic deformities being more common. Treatment primarily focuses on individualized approaches, supported by surgical interventions to alleviate clinical symptoms and enhance the patient's quality of life. Moving forward, there is also a need to further explore the impact of porencephalic malformations on neural development and behavior.

## Data Availability

The original contributions presented in the study are included in the article/Supplementary Material, further inquiries can be directed to the corresponding author.
